# Alzheimer Dementia Among Individuals With Down Syndrome

**DOI:** 10.1001/jamanetworkopen.2024.35018

**Published:** 2024-09-23

**Authors:** Eric Rubenstein, Salina Tewolde, Amy Michals, Jennifer Weuve, Juan Fortea, Matthew P. Fox, Marcia Pescador Jimenez, Ashley Scott, Yorghos Tripodis, Brian G. Skotko

**Affiliations:** 1 Boston University School of Public Health, Boston, Massachusetts; 2 Sant Pau Memory Unit, Neurology Department, Hospital de la Santa Creu i Sant Pau, Biomedical Research Institute Sant Pau, Universitat Autònoma de Barcelona, Spain; 3 Massachusetts General Hospital, Boston

## Abstract

**Importance:**

With the advancement in administrative data as a research tool and the reliance on public health insurance for individuals with Down syndrome, population-level trends in Alzheimer dementia in this population are beginning to be understood.

**Objective:**

To comprehensively describe the epidemiology of Alzheimer dementia in adults with Down syndrome in a full US Medicare and Medicaid sample.

**Design, Setting, and Participants:**

This cohort study included 132 720 adults aged 18 years or older with Medicaid and/or Medicare claims data with an *International Statistical Classification of Diseases and Related Health Problems* code for Down syndrome. Data were collected from January 1, 2011, to December 31, 2019, and analyzed from August 2023 to May 2024.

**Main Outcomes and Measures:**

The main outcome was prevalence of Alzheimer dementia in each calendar year and during the 9-year period. Alzheimer dementia incidence rates by calendar year and age and stratified for race or ethnicity as well as time to death after Alzheimer dementia diagnosis were also assessed.

**Results:**

There were 132 720 unique adults with Down syndrome from 2011 to 2019: 79 578 (53.2%) were male, 17 090 (11.7%) were non-Hispanic Black, 20 777 (15.7%) were Hispanic, 101 120 (68.8%) were non-Hispanic White, and 47 692 (23.3%) had ever had an Alzheimer dementia diagnosis. Incidence was 22.4 cases per 1000 person-years. The probability of an incident Alzheimer dementia diagnosis over 8 years was 0.63 (95% CI, 0.62-0.64) for those entering the study between ages 55 to 64 years. Mean (SD) age at incident diagnosis was 54.5 (7.4) years and median (IQR) age was 54.6 (9.3) years. Mean (SD) age at death among those with Alzheimer dementia was 59.2 (6.9) years (median [IQR], 59.0 [8.0] years). The mean (SD) age at onset for the Hispanic group was 54.2 (9.2) years, 52.4 (7.8) years for the American Indian or Alaska Native group, and 52.8 (8.2) years for the mixed race groups compared with 55.0 (7.8) years for the White non-Hispanic group. For age at death, there were no differences by sex. The mean (SD) age at death was later for the White non-Hispanic group (59.3 [6.8] years) compared with the Hispanic group (58.5 [7.8] years), Native American group (57.8 [7.1] years), and mixed race group (58.2 [7.0] years).

**Conclusions and Relevance:**

In this cohort study of adults with Down syndrome who were enrolled in Medicaid and Medicare, Alzheimer dementia occurred at high rates. Consistency with clinical studies of dementia in Down syndrome supports the use of administrative data in Down syndrome–Alzheimer dementia research.

## Key Points

**Question:**

What is the incidence and prevalence of Alzheimer dementia in patients with Down syndrome using data from the US Medicare and Medicaid?

**Findings:**

In this cohort study of 132 720 adults with Down syndrome enrolled in Medicaid or Medicare between 2011 to 2019, 23.3% of adults had Alzheimer dementia diagnoses, and the mean age of death was 59.2 years.

**Meaning:**

These findings suggest that Alzheimer dementia is almost universal among people with Down syndrome, and administrative claims data may offer valuable insights into improving care for this diverse population.

## Teaser

This cohort study examines the prevalence and incidence of Alzheimer disease among individuals with Down syndrome using US Medicare and Medicaid claims data.

## Introduction

Down syndrome is caused by the triplication of chromosome 21 and is the leading genetic cause of intellectual disability.^1^ With development of treatments for co-occurring conditions (eg, congenital heart defects)^1^ and advances in disability rights (eg, deinstitutionalization),^2^ there has been a dramatic increase of life expectancy for people with Down syndrome.^3^ Therefore, more people with Down syndrome are entering mid- and late adulthood and are experiencing chronic conditions associated with aging.

Alzheimer dementia occurs more often in people with Down syndrome compared with the general population^4,5^ because the amyloid precursor protein gene is located on the triplicated chromosome 21.^6^ By age 40, nearly all people with Down syndrome exhibit neuritic plaques and neurofibrillary tangles, the neuropathological hallmarks of Alzheimer disease; however, a subset will not manifest clinical symptoms.^5^ Timing of diagnosis after onset can be variable as there are no established reference standard diagnostic assessments of Alzheimer dementia in people with Down syndrome.

With advancements in methods to use administrative data for research,^7^ and the reliance on public health insurance for the Down syndrome population,^8^ we are now able to assess epidemiology of Alzheimer dementia in Down syndrome at the population level.^9^ Of particular note is the assessment of occurrence by race and ethnicity, as people with Down syndrome from racially and ethnically minoritized groups appear to experience worse health outcomes.^10^ To our knowledge, there has not yet been a thorough examination of Alzheimer dementia in Down syndrome at the level of a national health system. Our objective was to comprehensively describe the epidemiology of Alzheimer dementia in Down syndrome in a full US Medicare and Medicaid sample. We examined prevalence and incidence, and how both varied over time by race or ethnicity.

## Methods

This cohort study follows the Strengthening the Reporting of Observational Studies in Epidemiology (STROBE) reporting guideline (eAppendix 1 in Supplement 1). This study was determined to be non–human participants research by the institutional review board at Boston University Medical Campus, and informed consent was not required.

We used data from the Down Syndrome Toward Optimal Treatments and Health Equity using Medicaid Analytic eXtract study. Data included all adults with Down syndrome enrolled in Medicaid, Medicare (including Medicare Advantage), or both programs (dual enrolled) between 2011 to 2019. Medicaid is the publicly funded health insurer for many disabled people or people with low incomes or assets. Medicare provides insurance for nearly all adults aged 65 years and older, as well as some younger people with disabilities. Analyses were conducted in the summer and fall of 2023.

### Inclusion and Exclusion Criteria

We included all adult enrollees in Medicaid and/or Medicare who were enrolled for at least 1 year between 2011 to 2019 and met Down syndrome identification criteria. We excluded individuals with an age younger than 35 years at study completion, which we presumed unlikely for Alzheimer dementia in Down syndrome (2.2% of all with dementia claims) and those with recorded ages older than 80 years (0.8%).

### Down Syndrome and Alzheimer Dementia Identification

Down syndrome was identified by assessing an individual’s claims for any 1 inpatient or 2 outpatient *International Statistical Classification of Diseases and Related Health Problems, Ninth Revision* (*ICD-9*) or *Tenth Revision* (*ICD-10*) codes for Down syndrome (eAppendix 2 in Supplement 1). We used 2 established US Centers for Medicare & Medicaid Services Chronic Condition Warehouse algorithms to identify individuals that meet diagnostic criteria for Alzheimer disease (eAppendix 3 in Supplement 1).^11^ Although the *ICD-9* and *ICD-10* terminology refers to Alzheimer disease, these diagnoses represent diagnoses of dementia believed to be due to Alzheimer disease (eg, Alzheimer dementia, which is referred to as Alzheimer dementia throughout this article). The published algorithm scans claims over an interval of 3 years, meaning an enrollee would need to have 3 years of continuous enrollment to be considered eligible. The interval represents what is believed to be sufficient time for an enrollee who has dementia to accrue enough clinical encounters for a diagnosis to occur. Since individuals with Down syndrome were enrolled in our cohort a mean (SD) of 6.6 (2.7) years and median (IQR) 7.9 (4.4) years with higher annual health care use (205 claims per year compared with 52 claims per year in those without Down syndrome),^8^ we reduced our scanning interval to 1 year of enrollment. Using a shorter claim scanning interval better aligns with the experience of Alzheimer dementia in the Down syndrome population considering the high service use and potential for death from Alzheimer dementia before 3 years.^12^

### Other Variables

Although context and categories varied by state, enrollees reported race and ethnicity. Medicaid or Medicare classified responses and harmonized across states. Then, we aligned the Medicaid and Medicare classification to the Research Triangle Institute race coding system^13^ of American Indian or Alaskan Native, Asian or Pacific Islander, Black, Hispanic, White, and mixed race. All groups were mutually exclusive. Missing race or ethnicity data (approximately 16%) were imputed using multiple imputation with zip code and other demographics as estimators. Race and ethnicity were collected because Race and ethnicity were collected because they are important demographic information needed to equitably administer the Medicare and Medicaid programs. We used the American Community Survey 5-year summary data file to calculate the percentage of each race category at the zip code level. These proportions and demographic data were used for imputing the missing race and ethnicity. We did 30 imputations of a model that included age, sex, disability eligibility, dual enrollment, and zip code-level race and ethnicity distribution.^8^ We did not have ethnicity information beyond Hispanic or non-Hispanic. We classified enrollment type as Medicaid only, Medicare only, or dual-enrolled. Date of death was reported in both Medicaid and Medicare data.

### Statistical Analysis

Data were collected from January 1, 2011, to December 31, 2019, and analyzed from August 2023 to May 2024. Calculations were conducted using SAS version 9.4 (SAS Software) and R version 4.4.0 (R Project for Statistical Computing). Tests were 2-sided, and statistical significance was set at *P* < .05.

#### Prevalence and Incidence

Prevalence of Alzheimer dementia was calculated as the number who met Alzheimer dementia criteria during each calendar year (from 2011 to 2019), as well as any time during the 9-year period, divided by the number of included enrollees during those periods. We assessed prevalence trends throughout the calendar year by age (36 to 44 years, 45 to 54 years, and 55 to 64 years) and sex. We qualitatively examined differences in race or ethnicity and enrollment type (Medicaid only, Medicare only, or dual-enrolled) in each age stratum for study year 2019 to understand patterns at the end of our study.

We restricted all incidence calculations to beneficiaries who did not have Alzheimer dementia at the relevant baseline. We excluded individuals who had any dementia claim in their first year of observation in our study period. Therefore, our first estimate of incidence was in 2012. The restriction does not impart a left-censor bias because we had a near full Down syndrome population covering the full age range over 9 years with little cohort or period effects. We calculated person time from the date of enrollment to first Alzheimer dementia claim, death, loss-to-follow up, or end of the study (December 31, 2019) and subtracted 1 to account for the year washout period. We calculated Alzheimer dementia incidence rates specific to calendar year and for race or ethnicity, age group, and enrollment type within calendar years. We constructed Kaplan-Meier curves on both age and calendar year scales to depict risk over a given period and risk over the life course. Because of our large sample size and the subsequent likelihood that null-hypothesis statistical testing would find statistically significant differences that were not clinically meaningful or relevant,^14^ we elected not to conduct statistical testing. We present 95% CIs to present variability within our data.

#### Time to Death

We calculated the percentage of those with Alzheimer dementia who died in our cohort and age at death for both prevalent and incident dementia cases. We examined time to death from incident Alzheimer dementia diagnosis among those with Alzheimer dementia. We plotted time to death using Kaplan-Meier curves.

#### Sensitivity Analyses

We compared Alzheimer dementia claims from Medicare to Alzheimer dementia claims in Medicaid among dual enrollees. Our sensitivity analyses assessed what prevalence would be if all enrollees had access to Medicare. Then, with those estimates we adjusted our estimates for misclassification in the Medicaid only sample and calculated the percent difference between our adjusted sample and original population. Additionally, we ran a post-hoc sensitivity analysis restricting to those who were younger than age 65 years at study entry (an extreme age for someone with Down syndrome) to examine how potential misclassification in that oldest age group may impact full sample incidence. Lastly, we examined the impact of our imputation on race and ethnicity by comparing the imputed sample to those without missing data.

## Results

Of 132 720 adults with Down syndrome enrolled in Medicaid and/or Medicare, 79 578 (53%) were male, 17 090 (12.9%) were Black individuals, 21 899 (16.5%) were Hispanic individuals, 101 120 (68.8%) were White individuals, and mean (SD) age at study start was 36.0 (14.6) years. Over the 9 years, 31 007 individuals (23.3%) had Alzheimer dementia at baseline or were newly diagnosed during follow-up and of those, 16 685 of 31 007 (53.8%) were incident cases (Table). Those with Alzheimer dementia were older and more likely to be White individuals compared with those without Alzheimer dementia. Most with Alzheimer dementia were dual enrolled in Medicaid and Medicare, while most without were in Medicaid only.

**Table.** Occurrence of Alzheimer Dementia Among People With Down Syndrome Enrolled in Medicaid and/or Medicare, 2011-2019

Characteristics	Patients, No. (%)		
	Ever AD (n = 31 007)	Incident AD (n = 16 685)^a^	No AD (n = 101 713)
Sex			
Male	16 697 (53.9)	8930 (53.5)	53 951 (53)
Female	14 310 (46.2)	7775 (46.6)	47 762 (47)
Age at first claim, y			
<35	651 (2.1)	579 (3.5)	66 630 (65.5)
35-44	5043 (16.3)	3553 (21.3)	18 204 (17.9)
45-54	14 855 (47.9)	8481 (50.8)	11 621 (11.4)
55-64	8811 (28.4)	3392 (20.3)	4125 (4.1)
≥65	1647 (5.3)	680 (4.1)	1133 (1.1)
Mean (SD)	51.8 (8.1)	50.0 (8.2)	31.2 (12.6)
Median (IQR)	51.8 (10.2)	50.0 (9.8)	28 (20)
Race or ethnicity			
American Indian or Alaskan Native	143 (0.5)	88 (0.5)	982 (1)
Asian or Pacific Islander	320 (1)	203 (1.2)	3119 (3.1)
Black	2571 (8.3)	1524 (9.2)	12 995 (13.1)
Hispanic	1847 (6)	1122 (6.8)	18 930 (19)
White	25 711 (83.4)	13 493 (81.4)	61 916 (62.2)
Mixed race^b^	239 (0.8)	142 (0.9)	1573
Missing^c^	176	113	2198
Region			
Northeast	8318 (26.9)	4124 (24.7)	19 803 (19.6)
West	4545 (14.7)	2596 (15.6)	23 822 (23.6)
South	9213 (29.7)	5292 (31.7)	35 612 (35.3)
Midwest	8854 (28.6)	4635 (27.8)	21 684 (21.5)
US Territories	74 (0.2)	35 (0.2)	766 (0.8)
Death			
Yes	17 128 (55.3)	7331 (43.9)	9145 (9)
No	13 879 (44.8)	9354 (56.1)	92 568 (91)
Age at death, y			
Mean (SD)	59.2 (6.9)	58.6 (7.2)	50.7 (13.7)
Median (IQR)	59.0 (8)	58.0 (9)	53.0 (18)
Enrollment^d^			
Medicaid only	2404 (7.8)	1799 (10.8)	58 224 (57.2)
Medicare only	2871 (9.3)	1608 (9.6)	9344 (9.2)
Dual enrolled	25 732 (83.1)	13 278 (79.6)	34 145 (33.6)
Medicaid person-years^e^			
Mean (SD)	5.8 (3.2)	5.7 (3.1)	4.6 (3.1)
Median (IQR)	6.0 (6)	6.0 (6)	4.1 (6.1)
Ever Medicare person-years^e^			
Mean (SD)	30.1 (10)	29.1 (10.1)	18.0 (11.6)
Median (IQR)	32.0 (14)	30.0 (15)	17.0 (19)
Observed Medicare person-years^e^			
Mean (SD)	6.6 (2.7)	7.7 (1.9)	7.4 (2.5)
Median (IQR)	7.9 (4.4)	9.0 (2.4)	9.0 (2.9)

Abbreviation: AD, Alzheimer dementia.

^a^ Among all enrollees.

^b^ Those who chose multiple race categories.

^c^ Incident dementia indicated 1 years enrolled without claims.

^d^ Sixteen percent of race or ethnicity missing in the Medicaid data and imputed.

^e^ Because of stable enrollment, no minimum enrollment period was used.

### Prevalence

**Figure 1.**
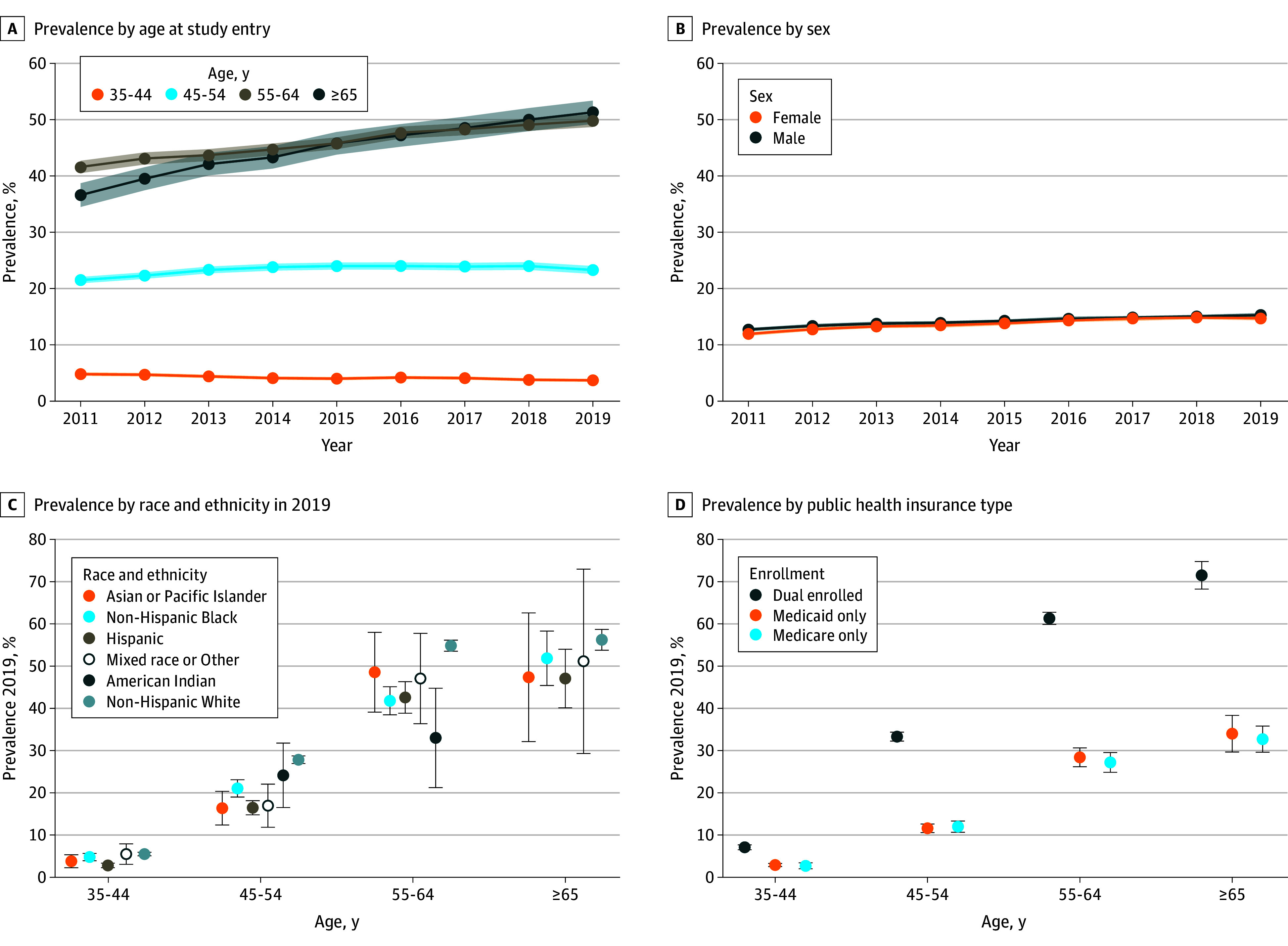
Prevalence of Alzheimer Dementia among People with Down Syndrome enrolled in Medicaid and/or Medicare, 2011-2019 Cells with values less than 10 are suppressed.

Between 2011 to 2019, yearly prevalence of Alzheimer dementia in the full sample ranged from 13.7% (95% CI, 13.4%-13.9%) to 15.3% (95% CI, 15.0%-15.5%). By age at cohort entry (Figure 1A), prevalence increased over time for those aged 55 to 64 years (5523 [41.6%] in 2011 to 7653 [49.8%] in 2019) and the 65 years or older group (1161 [42.1%] in 2011 to 2353 [51.3%] in 2019). There were no differences by sex (Figure 1B). By race or ethnicity in 2019 for each age category (Figure 1C), the non-Hispanic White group had the highest prevalence, and the American Indian and Alaskan Native group had the lowest prevalence, even though the sample size was small. Alzheimer dementia was most prevalent among individuals who were dual enrolled in both Medicaid and Medicare in all age groups (Figure 1D). In total, 7074 (12.2%) were discordant comparing Medicare and Medicaid claims. We found that adjusting for misclassification would increase prevalence estimates of Alzheimer dementia in those 35 to 44 years by 1.1% (4985 adjusted compared with 5043) and increase the 45 to 54 years (14 921 adjusted compared with 14 855) and 55 to 64 years groups by 0.5% (8213 adjusted compared with 8111) (eTable 1 in Supplement 1).

### Incidence

**Figure 2.**
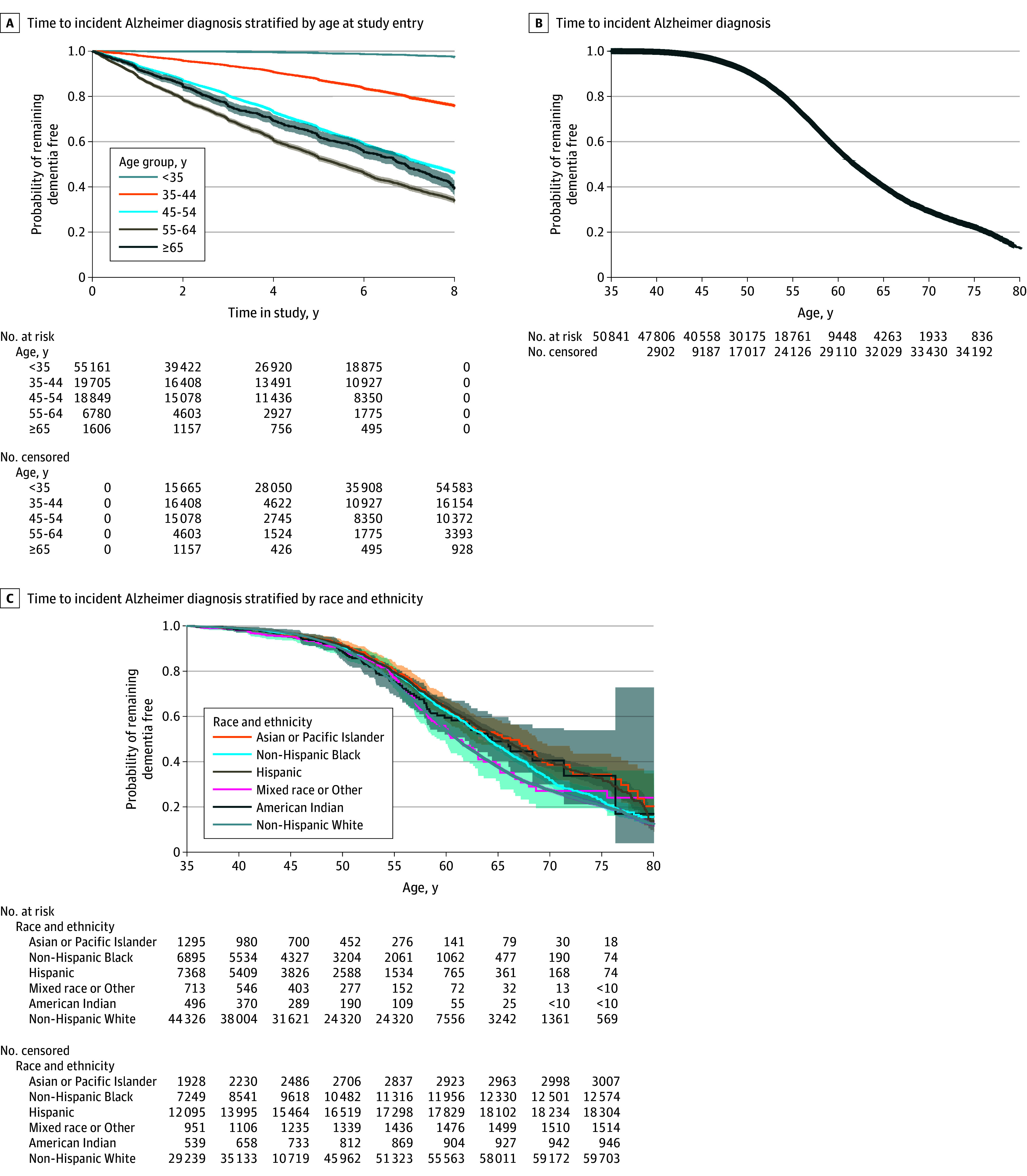
Kaplan-Meier Curves for Years to Incident Alzheimer Dementia Diagnosis in Medicaid and Medicare Enrolled Adults With Down Syndrome Cells with values less than 10 are suppressed.

Incidence rates for Alzheimer dementia in our full population was 22.4 cases per 1000 person-years in 2012 and 19.0 cases per 1000 person-years in 2019 (eTable 2 in Supplement 1). We found that an individual with Down syndrome who entered our cohort between ages 55 to 64 years would have a 63% (95% CI, 62%-64%) probability of receiving an Alzheimer dementia diagnosis if they were enrolled for 8 years (Figure 2A). When using age as our time axis, we saw that if an individual was to reach age 75 years, the probability of Alzheimer dementia diagnosis was near 0.8 (Figure 2B), accounting for death and loss to follow up. Rates were qualitatively similar for racial and ethnic groups until around 50 years where White non-Hispanic and Native American adults had a steeper decline in time without Alzheimer dementia compared with other groups (Figure 2C). In sensitivity analyses restricting to those entering the study before age 65 years (2780 were excluded [2.1%]) cumulative probability approached 0.88 by age 75 years (eFigure 1 in Supplement 1).

### Age at Diagnosis

**Figure 3.**
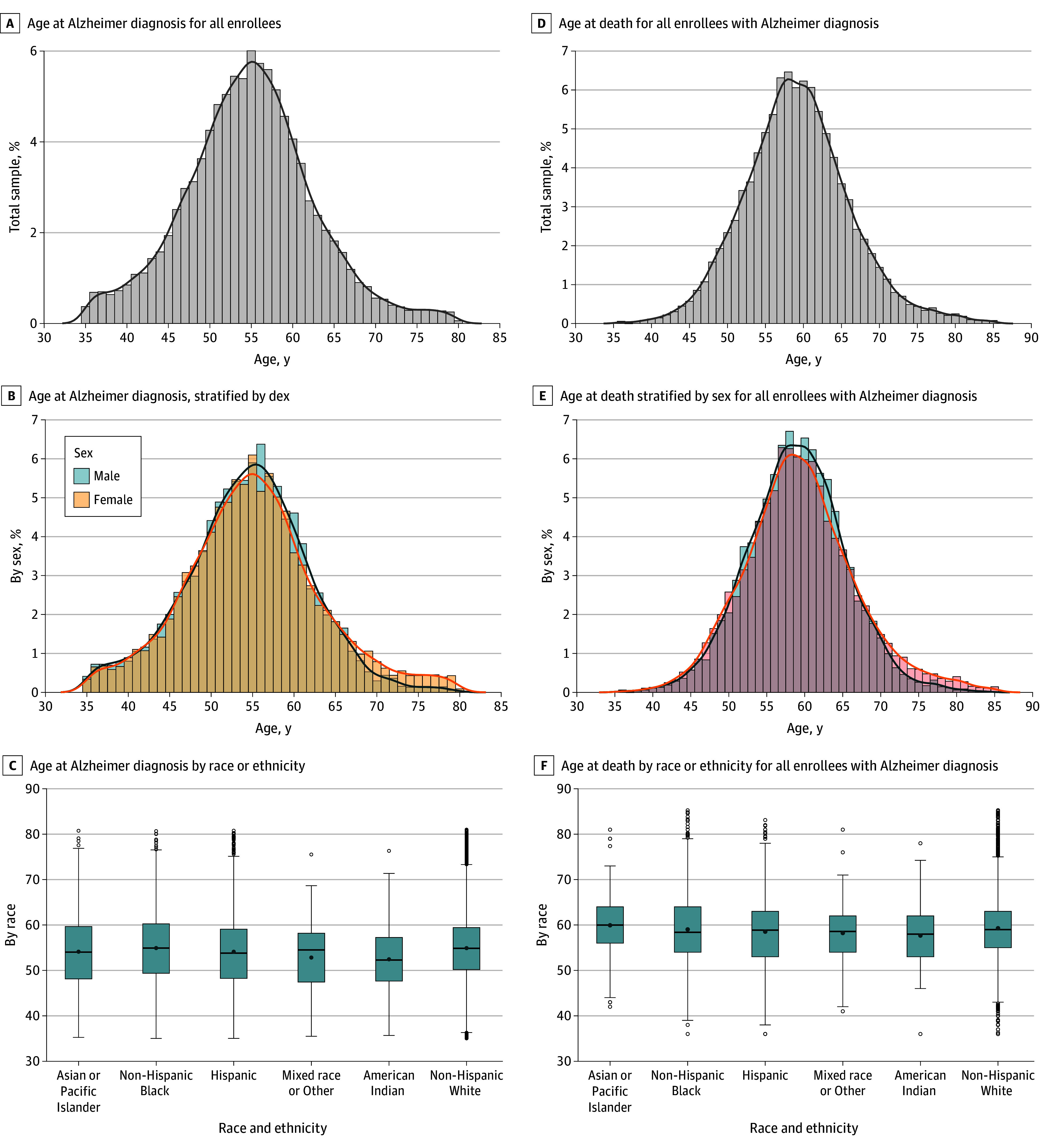
Age at Alzheimer Diagnosis and Death With Alzheimer Dementia in Medicaid and Medicare Enrolled Adults With Down Syndrome, 2011-2019

Age at incident diagnosis was normally distributed with mean (SD) age of 54.5 (7.4) years and median (IQR) age of 54.6 (9.3) years (Figure 3**A** and eTable 3 in Supplement 1). There were no clinically meaningful differences by sex (Figure 3B). By race or ethnicity (Figure 3C), there was no clinically meaningful difference between White and Black race, but mean (SD) age at incident diagnosis was earlier for Hispanic (54.2 [9.2] years), American Indian and Alaskan Native (52.4 [7.8] years), and mixed race (52.8 [8.2] years) groups compared with the non-Hispanic White group (55.0 [7.8] years). We saw minimal difference when restricting to those without missing race data (eTable 4 in Supplement 1).

### Mortality

**Figure 4.**
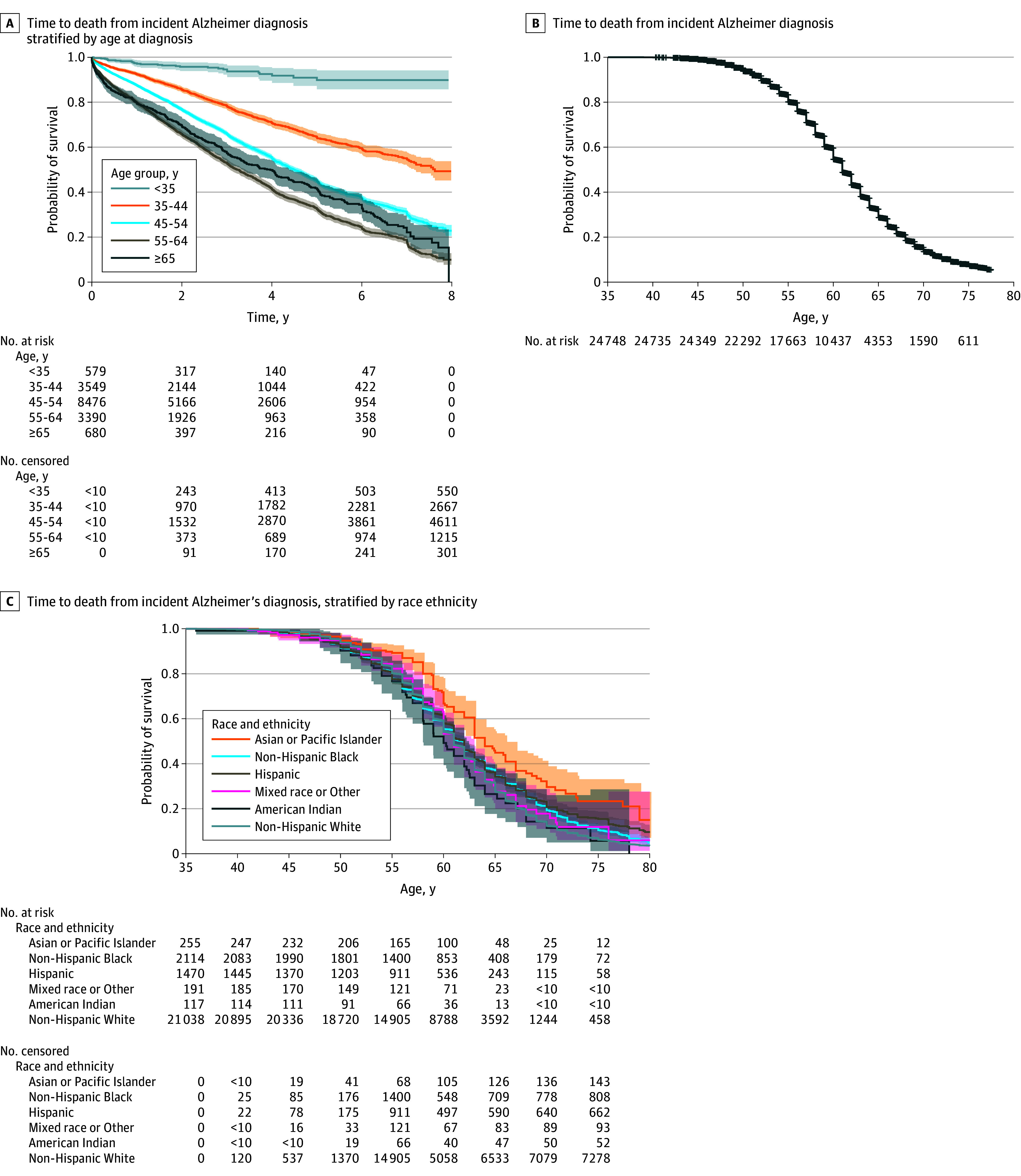
Kaplan-Meier Curves for Time From Alzheimer Dementia Diagnosis to Death for Adults With Down Syndrome Cells with values less than 10 are suppressed.

For those with any Alzheimer dementia mean age at death among those that died was 59.2 (6.9) years with a median (IQR) of 59.0 (8.0) (Figure 3D and eTable 3 in Supplement 1). Mean (SD) age among those who died without Alzheimer dementia was 50.7 (13.7) years (eTable 3 in Supplement 1). There were no differences by sex (Figure 3E). By race or ethnicity (Figure 3F), age at death was 0.8 years later for the non-Hispanic White group (59.3 [6.8] years) compared with the Hispanic group (58.5 [7.8] years). American Indian and Alaskan Native (57.8 [7.1] years) and mixed race (58.2 [7.0] years) groups had younger age at deaths compared with the non-Hispanic White group. Median time to death for the group that started the study age 55 to 64 years died within 3.3 years (Figure 4**A**). The median age at death for the population with Alzheimer dementia was 61.0 (55-72) years (Figure 4B). Survival probability across age (Figure 4C) was similar for Black, Hispanic, non-Hispanic White, and mixed race groups, while the Asian and Pacific Islander group had higher survival probability compared with other groups and American Indian and Alaskan Native had the lowest survival probability.

## Discussion

In this cohort study of adults with Down syndrome enrolled in Medicaid and Medicare, Alzheimer dementia was highly prevalent and incident. There were no differences between Black and White races and data were consistent with results from clinical studies. With the administrative data as a tool for research, and the reliance on public health insurance for the Down syndrome population we leveraged existing public health insurance systems to quantify occurrence and understand patterns of Alzheimer dementia in Down syndrome in a near full population sample.

### Epidemiology of Alzheimer Dementia

At the aggregate population level in 1 year, approximately 13% of adults older than 18 years with Down syndrome have Alzheimer dementia. Most with Alzheimer dementia were enrolled in Medicaid and Medicare as the group enrolled in Medicaid alone was considerably younger and few are solely enrolled in Medicare.^15^ Being dual enrolled allows for more health care service and use^15^ so it is also probable that there was more opportunity for Alzheimer dementia ascertainment in that group. There were regional differences but those are likely confounded by age, race, and service use characteristics between states. There was a slight increase in prevalence over time, which may reflect an increased life expectancy or a shifting age distribution for the Down syndrome population.^16^ Mean age of onset was in the early to mid-50s, which aligns with a meta-analysis of the literature, which found mean age of diagnosis to be 53.8 years.^17^ The finding that there was a lower hazard for the oldest age group may reflect a survival bias, where the group that survives dementia free to that time point has a different risk profile than those that do not survive that long. Alternatively, this older group may receive differential poorer care that increases probability of misclassification (eg, do not receive Alzheimer dementia diagnosis because they do not have health care clinicians that recognize the condition in Down syndrome).

Clinical studies have found more than 80% of adults aged 65 years and older with Down syndrome have symptoms of Alzheimer dementia.^18,19^ The RAND Institute estimated that prevalence reached 64% in adults aged older than 65 years,^20^ similar to our findings. The discrepancy may be due to our reliance on claims for Alzheimer dementia, which is passive and dependent on health care use. The hypothesis that Alzheimer dementia approaches 100% penetrant in people with Down syndrome is supported by the high prevalence we see in our data^21^; yet, there is a small proportion who will not exhibit symptoms if they survive into their late 60s or 70s.

Our estimates of life expectancy post-Alzheimer diagnosis were in line with a recent meta-analysis that included 550 individuals and found mean age of death of 58.4 (95% CI, 57.2-59.7) years and a mean duration of disease of 4.6 (95% CI, 3.7-5.5) years.^17^ The onset and duration in the Down syndrome population is much earlier and shorter compared with peers after adjusting for age.^22^ Our findings support the hypothesis that Alzheimer dementia imposed a limit on survival for adults with Down syndrome^17^ and that the gains in life expectancy for this population may stall until successful treatments and therapies are developed.

### Race or Ethnicity

Individuals with Down syndrome from minoritized racial and ethnic groups tend to have worse outcomes than their non-Hispanic White peers.^17,23^ For Alzheimer dementia, Black and non-Hispanic White individuals had similar mean ages of onset and age at death. It is possible that the biology of Alzheimer dementia in Down syndrome outweighs some of the disparity in Alzheimer dementia timing and mortality seen in the general population.^24^ Or, this similarity may reflect a survival paradox in which mortality rates in childhood are higher among Black individuals than among White individuals^17,23^ leading to worse health and mortality outcomes in the non-Hispanic White group compared with the non-Hispanic Black group in adulthood. It could also be that Alzheimer dementia onset is earlier for minoritized racial or ethnic groups (as seen in the general population),^25^ yet they are diagnosed later because of lack of health care access and other disparity.^26^ American Indian and Alaskan Native, Hispanic, and mixed race groups had earlier onset and earlier age at death compared with the non-Hispanic White group. Differences may reflect that in the US minoritized groups are exposed to more social and environmental risk factors for Alzheimer dementia, such as socioeconomic status and exposure to air pollution.^27,28^

### Misclassification in Claims

Claims data are an imperfect tool for identifying Alzheimer dementia because they are reliant on correct diagnoses and access to health care.^12,29,30,31^ We saw that 2% of adults younger than 35 years at end of study had Alzheimer dementia claims, which has not been previously reported in the literature. This suggests some over diagnosis, possibly from clinicians who struggle to distinguish cognitive decline from intellectual disability. For those older than 35 years, difficulty in diagnosis can lead to delayed or lack of diagnosis.^21^ Our sensitivity analysis found minimal differences while adjusting for imperfect sensitivity and specificity when comparing Medicaid to Medicare, but there is still likely misclassification because of a lack of well validated diagnostic tools.

### Limitations

This study has limitations. Our data was clinician-identified Alzheimer dementia, rather than true presence of disease or actual onset. However, our estimates align with clinical cohort studies that conduct follow up of individuals.^21^ Alzheimer dementia claims have not been validated in Down syndrome. Ethnicity data were not collected at a more refined level than Hispanic or non-Hispanic. It is possible that some individuals with Down syndrome had private or no insurance and would not be captured in our data. Based on employment rates and disability, we expect very few individuals to not have any Medicaid and/or Medicare enrollment.

## Conclusions

Alzheimer dementia is emotionally and financially challenging for people with Down syndrome and their families and is a major cause of death in this population. Our finding of mean onset of Alzheimer dementia at 54.5 years and duration of disease of 4.6 years was consistent with clinical studies. Given this consistency, administrative data can and should complement clinical research as a tool for researchers to understand Alzheimer dementia to assess population needs. As pharmaceutical interventions for Alzheimer dementia advance,^32,33^ it is pivotal that the population of individuals with Down syndrome is included in clinical trials and eligible for novel medication treatments.

## Notes

Supplement 1. **eAppendix 1**. *International Classification of Diseases, Ninth Revision* (*ICD-9*) and Tenth Revision (*ICD-10*) Codes for Down Syndrome **eAppendix 2.** Algorithm for Alzheimer’s disease **eTable 1.** Bias Analysis Assessing Misclassification of Alzheimer’s Dementia in Medicaid Compared With Medicare **eTable2.** Incidence Rate by Year and Demographics Among Adults With Down Syndrome **eTable 3.** Age at Incident Alzheimer Dementia and Age at Death With Alzheimer’s Dementia in Medicaid and Medicare Enrolled Adults With Down Syndrome, 2011-2019 **eTable 4.** Assessment of Imputation For Missing Race Among Medicaid Enrollees With Down Syndrome **eFigure 1.** Alzheimer Dementia Incidence Among Those With Down Syndrome by Whether We Restrict to Those Entering the Study Younger Than Age 65 Years **eFigure 2.** Age Distribution Comparing Age at Death For Adults With Down Syndrome With and Without Alzheimer Dementia

Supplement 2.
Data Sharing Statement
