# Supplementary material for: Alzheimer Dementia Among Individuals With Down Syndrome
Source: JAMA Netw Open. 2024 Sep 23;7(9):e2435018. doi: 10.1001/jamanetworkopen.2024.35018 (PMC11420697; doi:10.1001/jamanetworkopen.2024.35018)
Supplement: Supplement 1. — eAppendix 1. International Classification of Diseases, Ninth Revision (ICD-9) and Tenth Revision (ICD-10) Codes for Down Syndrome eAppendix 2. Algorithm for Alzheimer’s disease eTable 1. Bias Analysis Assessing Misclassification of Alzheimer’s Dementia in Medicaid Compared With Medicare eTable2. Incidence Rate by Year and Demographics Among Adults With Down Syndrome eTable 3. Age at Incident Alzheimer Dementia and Age at Death With Alzheimer’s Dementia in Medicaid and Medicare Enrolled Adults With Down Syndrome, 2011-2019 eTable 4. Assessment of Imputation For Missing Race Among Medicaid Enrollees With Down Syndrome eFigure 1. Alzheimer Dementia Incidence Among Those With Down Syndrome by Whether We Restrict to Those Entering the Study Younger Than Age 65 Years eFigure 2. Age Distribution Comparing Age at Death For Adults With Down Syndrome With and Without Alzheimer Dementia [file jamanetwopen-e2435018-s001.pdf]

# Supplemental Online Content

Rubenstein E, Tewolde S, Michals A, et al. A population study of Alzheimer dementia in Down syndrome . *JAMA Netw Open*. 2024;7(9):e2435018.
doi:10.1001/jamanetworkopen.2024.35018

**eAppendix 1**. *International Classification of Diseases, Ninth Revision* (*ICD-9*) and *Tenth Revision* (*ICD-10*) Codes for Down Syndrome
**eAppendix 2.** Algorithm for Alzheimer's disease
**eTable 1.** Bias Analysis Assessing Misclassification of Alzheimer's Dementia in Medicaid Compared With Medicare
**eTable2.** Incidence Rate by Year and Demographics Among Adults With Down Syndrome
**eTable 3.** Age at Incident Alzheimer Dementia and Age at Death With Alzheimer's Dementia in Medicaid and Medicare Enrolled Adults With Down Syndrome, 2011-2019
**eTable 4.** Assessment of Imputation For Missing Race Among Medicaid Enrollees With Down Syndrome
**eFigure 1.** Alzheimer Dementia Incidence Among Those With Down Syndrome by Whether We Restrict to Those Entering the Study Younger Than Age 65 Years
**eFigure 2.** Age Distribution Comparing Age at Death For Adults With Down Syndrome With and Without Alzheimer Dementia

This supplemental material has been provided by the authors to give readers additional information about their work.

© 2024 Rubenstein E et al. *JAMA Network Open.*

eAppendix 1. *International Classification of Diseases, Ninth Revision* (*ICD-9*) and *Tenth Revision* (*ICD-10*) Codes for Down Syndrome

| *ICD-9* | *ICD-10* |
|---|---|
| 758 | Q90 |
| | Q90.0 |
| | Q90.1 (Mosaic Down syndrome) |
| | Q90.2 (Translocation) |
| | Q90.9 (Unspecified) |

eAppendix 2. Algorithm For Alzheimer's Disease

From https://www2.ccwdata.org/web/guest/condition-categories-chronic

| Valid ICD-9 Codes | Valid ICD-10 codes | Number / Types of claims |
|---|---|---|
| 331.0, 331.11, 331.19, 331.2, 331.7, 290.0, 290.10, 290.11, 290.12, 290.13, 290.20, 290.21, 290.3, 290.40, 290.41, 290.42, 290.43, 294.0, 294.10, 294.11, 294.20, 294.21, 294.8, 797 (any DX on the claim) | F01.50, F01.51, F02.80, F02.81, F03.90, F03.91, F04, F05, F06.1, F06.8, G13.8, G30.0, G30.1, G30.8, G30.9, G31.01, G31.09, G31.1, G31.2, G94, R41.81, R54 (any DX on the claim) | At least 1 inpatient, SNF, HHA, HOP, or Carrier claim with DX code |

SNF: skilled nursing facility;
HHA: home health agency
HOP: hospital outpatient.

© 2024 Rubenstein E et al. *JAMA Network Open.*

eTable 1. Bias Analysis Assessing Misclassification of Alzheimer Dementia in Medicaid Compared With Medicare

1a. Sensitivity and specificity calculation by age group

| | | | | |
|---|---|---|---|---|
| | Total | | | |
| **Age** | 35-54 | | | |
| | | **Medicaid** | | |
| | | + | - | |
| **Medicare** | + | 18388 | 5982 | |
| | - | 1092 | 32738 | |
| | | | | |
| | **Sensitivity=** | 0.75 | **Specificity=** | 0.97 |
| | | | | |
| **Age** | 44-54 | | | |
| | | **Medicaid** | | |
| | | + | - | |
| **Medicare** | + | 2744 | 1136 | |
| | - | 261 | 9046 | |
| | **Sensitivity=** | 0.71 | **Specificity=** | 0.97 |
| | | | | |
| **Age** | 55-64 | | | |
| | | **Medicaid** | | |
| | | + | - | |
| **Medicare** | + | 9414 | 2786 | |
| | - | 511 | 6572 | |
| | **Sensitivity=** | 0.77 | **Specificity=** | 0.93 |
| **Age** | 65+ | | | |
| | | **Medicaid** | | |
| | | + | - | |
| **Medicare** | + | 5305 | 1617 | |
| | - | 234 | 1815 | |
| | | | | |
| | **Sensitivity=** | 0.77 | **Specificity=** | 0.89 |
| | | | | |

1b. Calculations for correction of misclassification in Medicaid

| **Age** | **Full N+** | **Full N-** | **No Medicaid N+** | **No Medicaid N-** | **Corrected N+** | **Corrected N-** | **%** |
|---|---|---|---|---|---|---|---|
| 35-44 | 5043 | 18204 | 4522 | 11865 | 4985 | 18261 | 1.14 |
| 45-54 | 14855 | 11621 | 13866 | 9039 | 14921 | 11554 | -0.45 |
| 55-64 | 8811 | 4125 | 8213 | 3157 | 8858 | 4077 | -0.53 |

Full= full Medicaid + Medicare Sample
No Medicaid= Cases and non cases with any Medicare claims
Corrected- Total cases we would expect correcting for imperfect sensitivity and specificity in Medicaid claims

© 2024 Rubenstein E et al. *JAMA Network Open.*

eTable 2. Incidence Rate by Year and Demographics Among Adults With Down Syndrome

| | **2012** | | **2013** | | **2014** | | **2015** | | **2016** | | **2017** | | **2018** | | **2019** | |
|---|---|---|---|---|---|---|---|---|---|---|---|---|---|---|---|---|
| | **PY** | 85 486 | **PY** | 88 513 | **PY** | 91846 | **PY** | 94292 | **PY** | 95 523 | **PY** | 95 818 | **PY** | 95 824 | **PY** | 94 757 |
| | N | Rate | N | Rate | N | Rate | N | Rate | N | Rate | N | Rate | N | Rate | N | Rate |
| **Any Alzheimer's dementia** | 2062 | 24.1 | 2120 | 24.0 | 2058 | 22.4 | 2195 | 23.3 | 2238 | 23.4 | 2105 | 22.0 | 2010 | 21.0 | 1798 | 19.0 |
| **Sex** | | | | | | | | | | | | | | | | |
| Male | 1140 | 24.9 | 1148 | 24.3 | 1105 | 22.6 | 1145 | 22.9 | 1191 | 23.5 | 1126 | 22.2 | 1034 | 20.4 | 990 | 19.9 |
| Female | 922 | 23.2 | 972 | 23.5 | 953 | 22.2 | 1050 | 23.8 | 1047 | 23.3 | 979 | 21.7 | 976 | 21.6 | 808 | 18.0 |
| **Age** | | | | | | | | | | | | | | | | |
| <35 | | | | | | | | | | | | | | | | |
| 35-44 | 206 | 11.6 | 204 | 11.4 | 163 | 9.0 | 201 | 11.0 | 221 | 12.0 | 199 | 10.7 | 164 | 8.8 | 183 | 9.6 |
| 45-54 | 886 | 44.4 | 906 | 45.4 | 856 | 43.1 | 852 | 43.3 | 851 | 44.4 | 814 | 43.9 | 736 | 41.2 | 593 | 34.8 |
| 55-64 | 811 | 94.4 | 800 | 83.4 | 850 | 80.4 | 917 | 80.4 | 950 | 79.1 | 872 | 70.3 | 893 | 70.8 | 797 | 63.3 |
| 65+ | 159 | 68.0 | 210 | 79.2 | 189 | 65.5 | 225 | 72.8 | 216 | 144.9 | 220 | 64.3 | 217 | 62.0 | 225 | 62.6 |
| Mean age, SD | 54.1 | 7.6 | 54.2 | 7.8 | 54.8 | 7.8 | 54.9 | 7.8 | 54.8 | 8.1 | 55 | 8.0 | 55.6 | 8.2 | 55.5 | 8.7 |
| Median age, SD | 54 | 10.0 | 54 | 10.0 | 55 | 9.0 | 55 | 9.0 | 55 | 9.0 | 55 | 9.0 | 55 | 9.0 | 56 | 10.0 |
| **Race ethnicity** | | | | | | | | | | | | | | | | |
| Non-Hispanic White | 1704 | 29.6 | 1748 | 29.6 | 1687 | 27.9 | 1772 | 29.0 | 1829 | 29.8 | 1670 | 27.4 | 1607 | 26.6 | 1397 | 23.5 |
| Black | 193 | 18.8 | 199 | 18.7 | 191 | 17.4 | 192 | 17.1 | 171 | 15.1 | 224 | 19.7 | 168 | 14.7 | 174 | 15.4 |
| Mixed race / Other | 11 | 9.0 | 14 | 10.8 | | | 22 | 14.7 | 28 | 18.3 | 19 | 12.2 | 26 | 16.7 | 13 | 8.3 |
| Asian / PI | 18 | 8.5 | 22 | 9.8 | 15 | 6.4 | 27 | 10.9 | 21 | 8.2 | 31 | 11.7 | 28 | 10.3 | 39 | 14.2 |
| Native | | | 10 | 13.1 | | | 15 | 18.1 | 18 | 21.5 | 10 | 11.8 | | | 13 | 15.3 |
| Hispanic | 114 | 9.0 | 114 | 8.4 | 132 | 9.2 | 152 | 9.8 | 152 | 9.5 | 140 | 8.5 | 165 | 9.8 | 149 | 8.7 |
| **Enrollment** | | | | | | | | | | | | | | | | |
| Medicaid only | 220 | 5.9 | 190 | 4.8 | 231 | 5.4 | 254 | 5.6 | 301 | 6.5 | 305 | 6.4 | 255 | 5.2 | 277 | 5.6 |
| Dual enrolled | 1579 | 45.0 | 1640 | 45.3 | 1588 | 41.9 | 1669 | 43.1 | 1727 | 44.5 | 1561 | 40.8 | 1528 | 40.4 | 1305 | 35.8 |
| Medicare only | 263 | 20.4 | 290 | 23.0 | 239 | 21.1 | 242 | 23.1 | 210 | 20.7 | 239 | 23.7 | 227 | 24.2 | 216 | 23.9 |

PY: Person years
PI: Pacific Islander
Rates per 1000 person years
Cells <10 are suppressed

© 2024 Rubenstein E et al. *JAMA Network Open.*

eFigure 1. Alzheimer Dementia Incidence Among Those With Down Syndrome by Whether We Restrict to Those Entering the Study Younger Than Age 65 Years

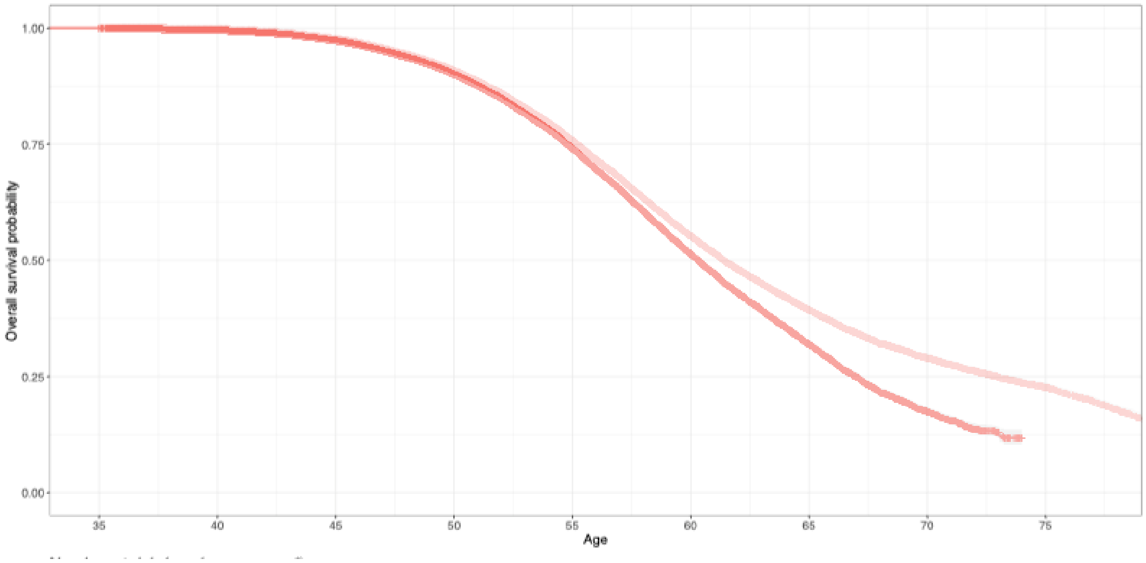


Darker red: restricted to those entering the study <= 65 years of age
Lighter red: full sample
N=2780 excluded; 2.1% of full sample

© 2024 Rubenstein E et al. *JAMA Network Open.*

eTable 3. Age at incident Alzheimer's dementia and age at death with Alzheimer's dementia in Medicaid and Medicare enrolled adults with Down syndrome, 2011-2019

| | **Age at incident Alzheimer's dementia** | | | **Age at death** | | |
|---|---|---|---|---|---|---|
| | **N** | **Mean (SD)** | **Median (IQR)** | **N** | **Mean (SD)** | **Median (IQR)** |
| **Total** | 16392 | 54.5 (7.4) | 54.6 (9.3) | 15369 | 59.2 (6.9) | 59.0 (8) |
| **Race / Ethnicity** | | | | | | |
| White | 13493 | 55 (7.8) | 54.9 (9.3) | 13074 | 59.3 (6.8) | 59.0 (8) |
| Black | 1 524 | 55 (8.8) | 54.9 (10.9) | 1221 | 59.0 (8.0) | 58.4 (10) |
| Hispanic | 1 122 | 54.2 (9.2) | 53.8 (10.9) | 731 | 58.5 (7.8) | 58.9 (10) |
| Mixed race | 142 | 52.8 (8.2) | 54.5 (10.7) | 97 | 58.2 (7.0) | 58.6 (8) |
| Asian / PI | 203 | 54.1 (9.2) | 54.0 (11.5) | 101 | 59.9 (7.7) | 60.0 (8) |
| Native | 88 | 52.4 (7.8) | 52.3 (9.6) | 63 | 57.8 (7.1) | 58.0 (9) |

SD: Standard deviation
IQR: Interquartile range

© 2024 Rubenstein E et al. *JAMA Network Open.*

eTable 4. Assessment of imputation for missing race among Medicaid enrollees with Down syndrome.

| | | | % w/ Alzheimer's dementia | | Age at Alzheimer's dementia | |
|---|---|---|---|---|---|---|
| | N after imputation | N not imputed | N after imputation | N not imputed | Imputed | Not imputed |
| White | 73803 | 59987 | 27.3 | 27.2 | 54.5 | 54.5 |
| Black | 13758 | 10259 | 15.6 | 14.3 | 54.5 | 54.4 |
| PI | 1036 | 261 | 13.4 | 9.3 | 53.6 | 56.0 |
| Asian | 3521 | 2092 | 7.9 | 8.5 | 54.4 | 51.7 |
| Native America | 841 | 795 | 10.2 | 10.6 | 51.6 | 51.5 |
| Multiple | 6573 | 1279 | 13.2 | 6.9 | 56.8 | 54.0 |

© 2024 Rubenstein E et al. *JAMA Network Open.*

eFigure. Age distribution comparing age at death for adults with Down syndrome with and without Alzheimer's dementia

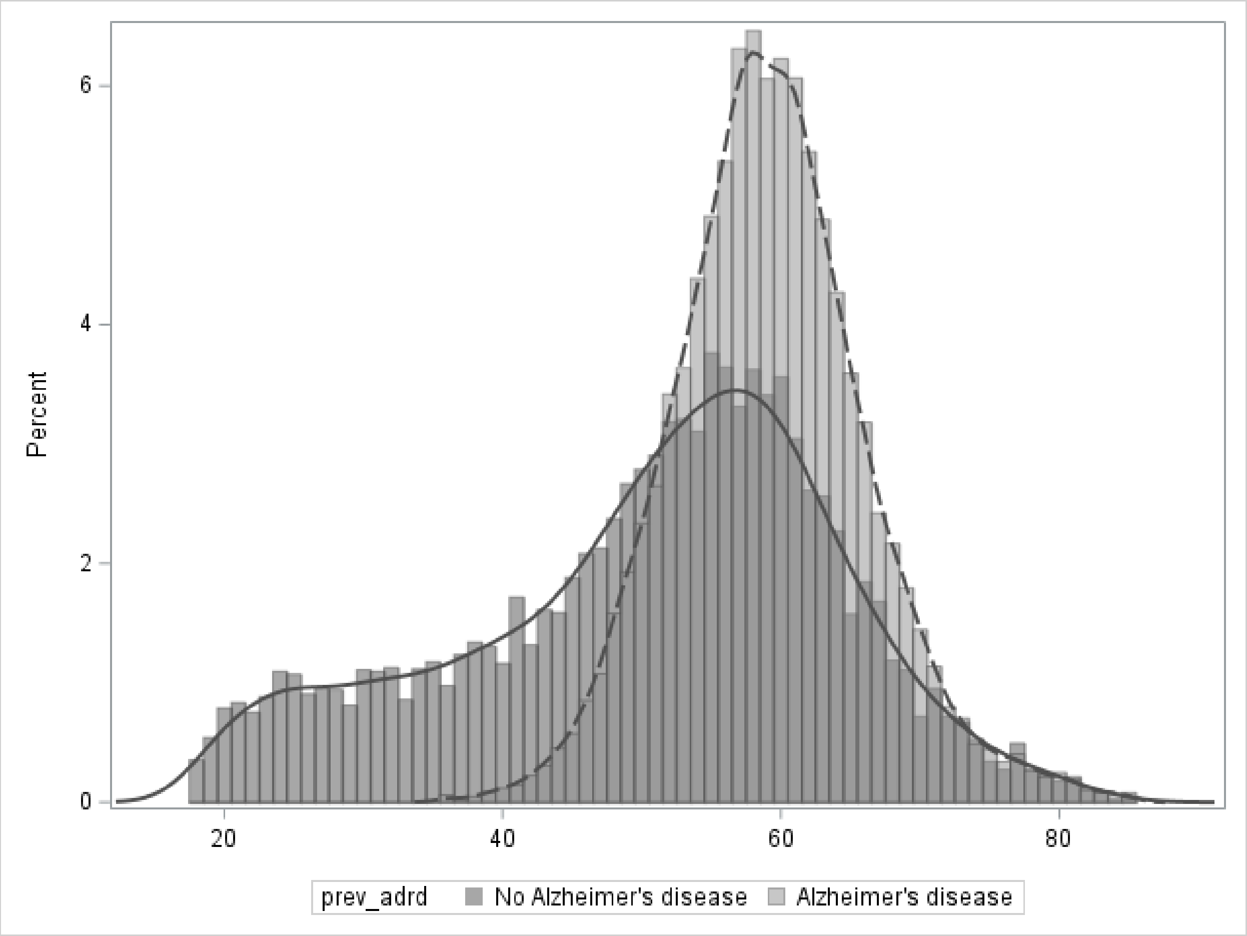


© 2024 Rubenstein E et al. *JAMA Network Open.*
