# Supplementary material for: Alzheimer Dementia Among Individuals With Down Syndrome
Source: JAMA Netw Open. 2024 Sep 23;7(9):e2435018. doi: 10.1001/jamanetworkopen.2024.35018 (PMC11420697; doi:10.1001/jamanetworkopen.2024.35018)
Supplement: Supplement 2. — Data Sharing Statement [file jamanetwopen-e2435018-s002.pdf]

Data Sharing Statement

Rubenstein. Alzheimer Dementia Among Individuals With Down Syndrome. *JAMA Netw Open*. Published September 23, 2024. doi:10.1001/jamanetworkopen.2024.35018

Data
Data available: No

Additional Information
Explanation for why data not available: Data are not able to be shared under a data use agreement with the Centers for Medicare and Medicaid Services.
